# PATIENT SATISFACTION WITH HOSPITAL-BASED OUTPATIENT REHABILITATION AFTER STROKE IN SWEDEN AND ITS ASSOCIATION WITH LIFE SATISFACTION AND HEALTH-RELATED QUALITY OF LIFE: A LONGITUDINAL NATIONAL REGISTER STUDY

**DOI:** 10.2340/jrm.v58.43966

**Published:** 2026-01-14

**Authors:** Anna BRÅNDAL, Britt-Marie STÅLNACKE, Gudrun M. JOHANSSON

**Affiliations:** 1 Department of Public Health and Clinical Medicine, Umeå University, Umeå; 2 Department of Community Medicine and Rehabilitation, Rehabilitation Medicine, Umeå University, Umeå, Sweden

**Keywords:** health, personal satisfaction, quality of life, stroke, outpatient rehabilitation

## Abstract

**Objective:**

To examine stroke survivors’ satisfaction with hospital-based outpatient rehabilitation and its association with life satisfaction and health-related quality of life (HRQoL), and whether sex and age affect possible associations.

**Design:**

A longitudinal national register study.

**Methods:**

Data from the Swedish national quality register were used and included 1,068 patients with stroke performing outpatient rehabilitation. Self-reported questionnaires collected on admission, discharge, and at 1-year follow-up were analysed. Regression analyses were used to assess possible associations between patient satisfaction and life satisfaction (Life Satisfaction Questionnaire, LiSat-11) and HRQoL (EuroQol Five Dimensions questionnaire, EQ-5D).

**Results:**

Over 71% of the included patients were satisfied with their rehabilitation process on discharge. Satisfied patients also reported higher scores on global LiSat-11 and higher EQ-5D values. Older patients (> 58 years) satisfied with their rehabilitation process were more likely to be satisfied with global LiSat-11. Women dissatisfied with the rehabilitation process had lower EQ-5D values on discharge.

**Conclusion:**

Patient satisfaction with hospital-based outpatient rehabilitation was associated with life satisfaction and HRQoL. Potential differences linked to sex and age support the importance of individually tailored rehabilitation strategies. Evaluating self-reported outcomes and experiences over time is essential for improving long-term recovery and for further development of person-centred stroke rehabilitation.

## LAY ABSTRACT

This study investigated satisfaction with rehabilitation, and how this relates to self-reported life satisfaction and health-related quality of life in 1,068 individuals with stroke in Sweden. Using data from a national quality register, we found that most patients were satisfied with their outpatient rehabilitation in hospital settings, both directly after the rehabilitation period and 1 year later. Those who were satisfied with the rehabilitation also reported better life satisfaction and better health-related quality of life. These relationships somewhat differed depending on sex and age, which highlights the importance of individually tailored outpatient rehabilitation after stroke.

Stroke is a common cause of disability in the adult population worldwide (1). Due to better treatment, growing numbers of people survive with stroke-related sequelae and need rehabilitation to function in everyday life (2–4). As stroke is more common in the older population, rehabilitation services are often designed for the elderly (5). Although people of working age represent a smaller proportion of the total stroke population, the number of working-age stroke survivors is expected to increase (6). Because they will live with their stroke-related consequences for a longer time, rehabilitation intervention is of critical importance (7). Previous research has shown that participation in work and social activities is linked to both Life Satisfaction (LS) and Health-Related Quality of Life (HRQoL) in this group (7, 8).

In Sweden, the duration of hospital stays for inpatient rehabilitation in general has declined, with a corresponding shift towards outpatient rehabilitation (9). For patients with stroke, early supported discharge in the subacute post-stroke phase is beneficial in patients treated in modern stroke units (10). Subsequent outpatient rehabilitation post-stroke is increasingly delivered by primary healthcare services and/or municipal care providers. In addition, stroke survivors of working age may be offered hospital-based outpatient rehabilitation to better facilitate ordinary life at home and at work. The National Board of Health and Welfare in Sweden provides guidelines for stroke care (2), which mainly address acute medical care and inpatient rehabilitation. To strengthen the scientific evidence regarding outpatient rehabilitation post-stroke, more research is needed in this area.

Important indicators to measure post-stroke outcome and the effect of rehabilitation include LS and HRQoL (11, 12). LS reflects an individual’s overall appraisal of life (13), whereas HRQoL captures the relationship between health, well-being, and the ability to function in physical, mental, and social domains (14). Patient-reported outcome measures (PROMs) are commonly used to assess aspects such as LS and HRQoL, whereas patient-reported experience measures (PREMs) capture patients’ perceptions of care processes, including satisfaction with rehabilitation (15). Previous studies have shown that many stroke survivors, particularly those of working age, report poor LS (16) and HRQoL (17). Despite some inconsistencies across studies, women tend to have poorer functional recovery and lower self-reported quality of life compared with men (18–21).

Age-related differences in recovery priorities have also been described, where younger individuals emphasize the importance of returning to work and social participation, while older individuals prioritize independence in daily activities (22, 23). Patient satisfaction with stroke rehabilitation has been explored using PREMs (11, 24, 25). However, the relationship between satisfaction with rehabilitation and outcomes such as LS or HRQoL remains insufficiently understood, particularly regarding potential differences depending on age and sex (20, 25–27).

The primary aim of this study was thus to investigate the effects of post-stroke hospital-based outpatient rehabilitation on LS and HRQoL before, immediately after, and at 1-year follow-up using PROMs from a national quality register. A secondary aim was to investigate whether the patients’ satisfaction with rehabilitation, assessed with a PREM, was associated with their LS or HRQoL, and if age and/or sex affected possible associations. We hypothesized that women would rate poorer LS and HRQoL. Additionally, we hypothesized that patients who were most satisfied with rehabilitation would also be satisfied with LS and report better HRQoL.

## METHODS

The register study was approved by the Regional Ethics Review board in Uppsala, Sweden (Dnr 2020-02355) and the administrator responsible for the register agreed to extraction of data.

### Register

SveReh is a national quality online register of rehabilitation medicine (previously named WebRehab Sweden) to which about 20 rehabilitation units in Sweden report (28). Stroke is one of the many diagnoses that are reported in the register (29). Patients are assessed at 3 time points: (*i*) on admission to the rehabilitation unit, (*ii*) on discharge from the rehabilitation unit, and (*iii*) at 1-year (± 2 months) follow-up from the onset date (if the onset date is missing or more than 9 months have passed between onset and admission, the interval is calculated based on the admission date instead) (28). The register contains patient data such as ICD codes, demographics, process measures, and outcomes measures including patient-reported responses.

### Study population

**Fig. 1:**
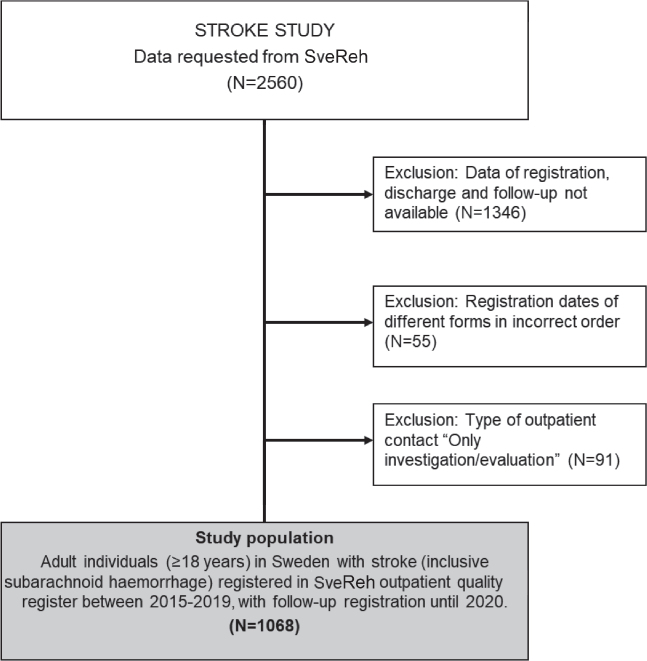
Flowchart of study population selection.

Eligible for this study were patients with stroke registered in SveReh between 2015 and 2020. The patients had been admitted for outpatient rehabilitation at 14 units nationwide. All had participated in multidisciplinary rehabilitation that included person-centred and task-oriented treatment according to national guidelines for stroke. Rehabilitation periods varied depending on the goals set out in their individual rehabilitation plan. Inclusion criteria for the present study were: (*i*) 18 years of age or older, and (*ii*) previous stroke (i.e., ischaemic stroke, intracerebral haemorrhage, or subarachnoid haemorrhage). Fig. 1 shows a flowchart of the inclusion procedure.

### Measurements

Baseline data included sex, age, stroke subtype, education, marital status, children in household, and vocational situation. Education level was categorized as compulsory school, upper secondary school, and university. In this study, the compulsory school categories “other” and “don’t know” were also added. Furthermore, marital status was categorized as single or living with a partner and vocational situation was defined as productive (> 50% working or studying), benefit (e.g., parental leave, > 50% sick leave) and retired.

The Life Satisfaction Questionnaire (Li-Sat-11) (22) is a patient-reported outcome measure (PROM) that is recommended for assessing LS after stroke (23). LiSat-11 includes 1 global item “Life as a whole” and 10 domain-specific items regarding vocation, economy, leisure, contacts with friends and acquaintances, sexual life, activities of daily living, family life, partner relationship, somatic health, and psychological health. Each item is scored on a Likert scale with 6 response levels: 1 = very dissatisfied, 2 = dissatisfied, 3 = rather dissatisfied, 4 = rather satisfied, 5 = satisfied, and 6 = very satisfied. In this study, only the global item was analysed and the scores for “Life as a whole” were dichotomized to separate those who were satisfied (scores 5–6) from those who were not satisfied (scores 1–4) (22).

The EuroQol five dimensions questionnaire (EQ-5D) (30) is the most used PROM of HRQoL after stroke (31, 32). EQ-5D consists of 5 dimensions: mobility, self-care, usual activities, pain/discomfort, and anxiety/depression. The EQ-5D 3 Level version is included in SveReh. This version contains 3 response levels: no problems, some problems, and extreme problems. The EQ-5D can be presented as a visual analogue scale (EQ VAS) allowing a direct valuation of the current state of health and a single utility value (EQ-5D index), reflecting population preferences. The Swedish tariff of the EQ-5D Index was used (33).

Patient satisfaction was assessed by a PREM that is included in SveReh. The questionnaire contains process-related items regarding the patient’s degree of satisfaction with (*i*) the attention received from the staff, (*ii*) the cooperation with the staff, (*iii*) the rehabilitation process, (*iv*) the patient’s influence over the rehabilitation process including the individual rehabilitation plan, (*v*) the information given concerning the stroke, (*vi*) the information given on where to get more support if needed after discharge, and (*vii*) the information and attention the family and relatives had received during the patient’s rehabilitation at the clinic. All 7 items are answered on discharge whereas 4 items (3, 4, 5, and 7) are answered at the 1-year follow-up. Each item is scored on a 4-point Likert scale: very dissatisfied, dissatisfied, satisfied, and very satisfied. In this study, the responses “dissatisfied” and “very dissatisfied” were merged into 1 response category due to the low number of responses in the latter. The response categories “satisfied” and “very satisfied” were kept separate.

### Statistical analysis

Crude data of the different questionnaires and of their coverage information are presented separately for admission, discharge, and follow-up, separated by sex as shhown in Supplementary material. Proportions of crude data of patient satisfaction variables and their change from discharge to follow-up are displayed in Sankey diagrams.

Descriptive data of the study population are presented in categories described earlier based on the baseline data registered on admission. The included patients who appeared several times in the data file were analysed in the study only for the first registered rehabilitation period. Data on global LS (life as a whole) were presented in proportions, and data on EQ-5D (VAS and index) were presented in mean and SD, separately for admission, discharge, and at 1-year follow-up, by sex.

To investigate the associations between patient satisfaction and global LS and EQ-5D on discharge and follow-up, regression models were used. The odds ratio (OR) for patient satisfaction and association with global LS was calculated with logistic regression models. Coefficients for patient satisfaction and association with EQ-5D were calculated with linear regression. The regression models were performed separately for discharge and follow-up, and both unadjusted and multivariable adjusted. The multivariable adjusted regression models included age on admission (in categories: 18–44, 45–49, 50–54, 55–59, 60–64, 65–69, 70 and above), sex, education level, stroke subgroup, and data of the outcome (categorized global LS variable, EQ-5D Index, and EQ VAS) on admission (for discharge analysis) or on discharge (if missing on discharge; data on admission were used for follow-up analysis). The 95% confidence intervals calculated by robust standard errors and the heteroscedasticity HC3 option were applied for the linear regression analysis.

To investigate possible effect modification by sex and age (below or above median age 57 yearson discharge and 58 years at follow-up) on the associations between patient satisfaction and global LS and EQ-5D, we calculated interaction between sex and age and the outcomes, in similar regression models to those described above, with separate analyses for sex and age. *P*-values were used to determine statistical significance for the categories included in the patient satisfaction questionnaire within the regression models. These were calculated as the two-sided significance level of the z-value (in logistic regression) and *t* statistics (in linear regression). All analyses were performed using Stata 17.0 MP (StataCorp LLC, College Station TX, USA).

## RESULTS

Characteristics of the 1,068 patients in the study are presented in Table I. The cohort consisted of 61% men (mean age 57 years) and 39% women (mean age 53 years). Most of the patients were aged 45–64 years (67%). Ischaemic stroke was the most common cause of stroke (71%). A higher proportion of women than men had suffered from subarachnoid haemorrhage (17% and 6%, respectively). Most of the patients lived with a partner (69%) and more women than men had children living at home (37% and 27%, respectively). Approximately one-third had a university education, and 16% of the patients were retired. The rehabilitation programme typically involved between 5 and 20 h per week of participation, with an average duration of about 10 weeks.

**Table I:** Baseline data of the study population

Item	Total *n* = 1,068	Women *n* = 417	Men *n* = 651
Age			
18–44 years	176 (16.5 %)	97 (23.3%)	79 (12.1%)
45–64 years	715 (66.9%)	259 (62.1%)	456 (70.0%)
65 years or older	177 (16.6%)	61 (14.6%)	116 (17.8%)
Stroke sub-groups			
Subarachnoid haemorrhage	111 (10.4%)	72 (17.3%)	39 (6.0%)
Ischaemic stroke	756 (70.8%)	265 (63.5%)	491 (75.4%)
Intracerebral haemorrhage	201 (18.8%)	80 (19.2%)	121 (18.6%)
Marital status			
Single	327 (30.6%)	129 (30.9%)	198 (30.4%)
Living with a partner	741 (69.4%)	288 (69.1%)	453 (69.6%)
Children at home			
Yes	334 (31.3%)	156 (37.4%)	178 (27.3%)
No	710 (66.5%)	252 (60.4%)	458 (70.4%)
Missing	24 (2.2%)	9 (2.2%)	15 (2.3%)
Education level			
Compulsory school (9 years)*	249 (23.3%)	90 (21.6%)	159 (24.4%)
Upper secondary school	496 (46.4%)	189 (45.3%)	307 (47.2%)
University	323 (30.2%)	138 (33.1%)	185 (28.4%)
Vocational situation			
Productive	180 (16.9%)	73 (17.5%)	107 (16.4%)
Benefit	675 (63.2%)	275 (65.9%)	400 (61.4%)
Retired	169 (15.8%)	58 (13.9%)	111 (17.1%)
Missing	44 (4.1%)	11 (2.6%)	33 (5.1%)
Rehabilitation			
Hours/week			
< 5	75 (7.0%)	31 (7.4%)	44 (6.8%)
5 to 10	384 (36.0%)	150 (36.0%)	234 (35.9%)
10 to 20	417 (39.0%)	151 (36.2%)	266 (40.9%)
20 to 30	105 (9.8%)	44 (10.6%)	61 (9.4%)
> 30	87 (8.2%)	41 (9.8%)	46 (7.1%)
Duration in days, mean (SD)	67.3 (43.9)	70.6 (45.4)	65.2 (42.8)

* Also includes categories “other” and “don’t know”.

The coverage rate for the various questionnaires administered on admission, discharge, and at 1-year follow-up ranged between 62% and 98%. The capture ratios of the EQ-5D, LiSat-11 and patient satisfaction in the studied population from each time point, separately for sex, are presented in Table SI. Crude values of the separate questionnaires are presented in Tables SII–SIV.

### Life satisfaction and health-related quality of life

A higher proportion of women reported lower scores on the global item “Life as a whole” (LS) and lower values on EQ VAS and EQ Index (HRQoL) compared with men across all time points (Table II). Both men and women exhibited higher LS on discharge, an increase that declined at the 1-year follow-up. In contrast, higher scores on HRQoL on discharge remained at the 1-year follow-up for both women and men.

**Table II:** Summarized data of Life Satisfaction and Health-Related Quality of Life, for admission, discharge, and 1-year follow-up, separately by sex

Item	Admission		Discharge		Follow-up	
	Women	Men	Women	Men	Women	Men
Life Satisfaction, *n* (%)	*n* = 261	*n* = 401	*n* = 297	*n* = 440	*n* = 339	*n* = 558
Not satisfied with “Life as a whole” (scores 1–4)	177 (67.8%)	253 (63.1%)	172 (57.9%)	226 (51.4%)	221 (65.2%)	319 (59.3%)
Satisfied with “Life as a whole” (scores 5–6)	84 (32.2%)	148 (36.9%)	125 (42.1%)	214 (48.6%)	118 (34.8%)	219 (40.7%)
Health-Related Quality of Life, mean (SD)	*n* = 367	*n* = 568	*n* = 338	*n* = 493	*n* = 407	*n* = 633
EQ5D Index	0.77 (0.12)	0.80 (0.12)	0.81 (0.12)	0.84 (0.11)	0.80 (0.12)	0.83 (0.12)
Self-reported health state (EQ VAS)	59.3 (16.9)	63.0 (19.4)	67.7 (18.3)	71.0 (17.4)	66.7 (19.0)	68.8 (18.7)

EQ-5D: EuroQol five dimensions questionnaire.

### Patient satisfaction

**Fig. 2:**
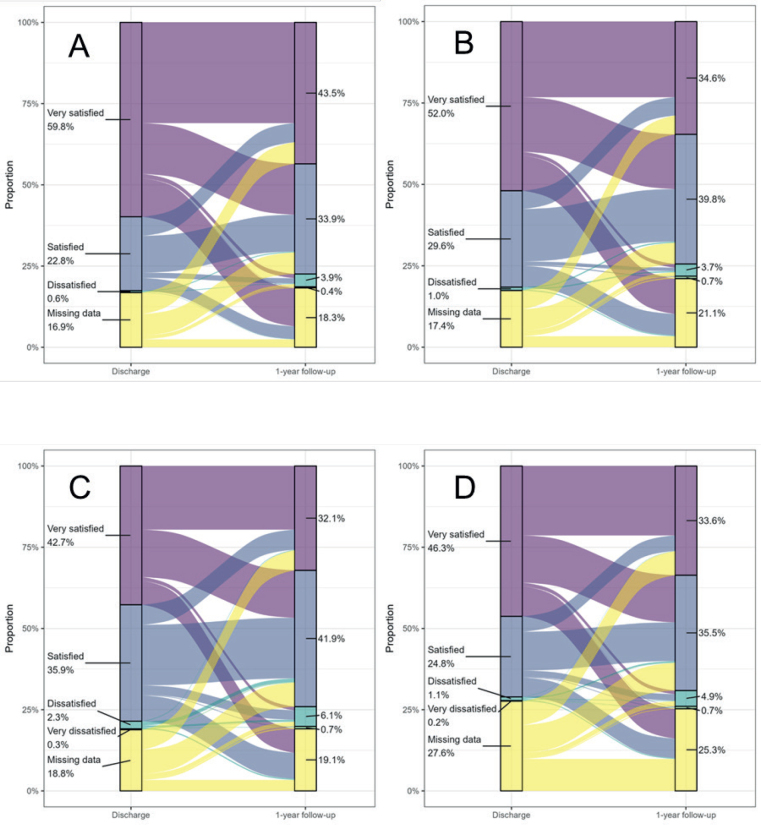
Sankey diagrams visualizing the changes of patient satisfaction from discharge to 1-year follow-up regarding 4 items: (A) rehabilitation process, (B) patient’s influence over the rehabilitation process, (C) information given concerning the stroke, and (D) information and attention the family and relatives had received.

Fig. 2 shows Sankey diagrams of patient satisfaction on discharge and at 1-year follow-up. On discharge, most patients (> 71 %) reported being satisfied or very satisfied. However, the proportion of patients who were very satisfied on discharge declined at the 1-year follow-up due to a change to increased proportions of satisfied individuals and dissatisfied individuals. Additionally, the rate of missing data showed a slight increase at the 1-year follow-up for all except the final one of the 4 items.

### Associations between patient satisfaction and life satisfaction and health-related quality of life on discharge and at 1-year follow-up.

Table III displays the regression models of patient satisfaction and association with LS and HRQoL on discharge and at follow-up. On discharge, patients who were very satisfied with the rehabilitation process were more likely to be satisfied with the global LS and reported higher values on EQ VAS. These associations continued to be significant at the 1-year follow-up. Furthermore, patients dissatisfied with the rehabilitation process reported lower values on EQ VAS, an association that was still significant at the 1-year follow-up and further accompanied by lower values on the EQ-5D Index. On discharge, patients very satisfied with the information provided about the stroke were more likely to be satisfied with the LS and reported better HRQoL. These associations were not significant at the 1-year follow-up except for the EQ VAS. On discharge, being very satisfied with staff cooperation was associated with being satisfied with LS and better HRQoL, while being very satisfied with the attention received from staff was associated with better HRQoL. Notably, satisfaction with one’s own influence over the rehabilitation process and satisfaction with the information and attention given to family and relatives were only associated with the LS and HRQoL at the 1-year follow-up.

**Table III:** Regression models of patient satisfaction and association with Life Satisfaction (life as a whole) and Health-Related Quality of Life (EQ-5D) on discharge and at 1-year follow-up

Item	Life as a whole^a^			EQ-5D Index^b^			EQ VAS^b^		
	Unadjusted	Multivariable adjusted	*p*-value	Unadjusted	Multivariable adjusted	*p*-value	Unadjusted	Multivariable adjusted	*p*-value
	OR (95% CI)	OR (95% CI)		Coefficient (95% CI)	Coefficient (95% CI)		Coefficient (95% CI)	Coefficient (95% CI)	
Discharge									
Rehabilitation process									
Very dissatisfied/dissatisfied	NA	NA		–0.08 (–0.22, 0.06)	–0.09 (–0.30, 0.12)	0.406	–17.29 (–35.68, 1.09)	**–29.48** (–46.13, –12.83)	0.001
Satisfied	1, ref	1, ref		0, ref	0, ref		0, ref	0, ref	
Very satisfied	2.01 (1.43, 2.84)	**2.09** (1.34, 3.24)	0.001	0.04 (0.02, 0.05)	**0.02** (0.00, 0.03)	0.011	7.81 (5.04, 10.59)	**4.79** (2.42, 7.16)	< 0.001
Patient’s influence over the rehabilitation process									
Very dissatisfied/dissatisfied	0.98 (0.21, 4.45)	0.36 (0.01, 10.55)	0.550	–0.02 (–0.15, 0.11)	–0.02 (–0.13, 0.09)	0.733	–10.30 (–25.19, 4.59)	–13.41 (–27.28, 0.45)	0.058
Satisfied	1, ref	1, ref		0, ref	0, ref		0, ref	0, ref	
Very satisfied	1.18 (0.87, 1.62)	1.24 (0.83, 1.85)	0.292	0.02 (0.00, 0.04)	0.01 (–0.00, 0.02)	0.148	3.58 (0.95, 6.21)	2.14 (–0.12, 4.40)	0.063
Information given concerning the stroke									
Very dissatisfied/dissatisfied	0.99 (0.41, 2.38)	0.77 (0.23, 2.56)	0.668	0.01 (–0.05, 0.06)	0.02 (–0.02, 0.06)	0.331	–2.08 (–11.25, 7.09)	–3.10 (–11.34, 5.14)	0.459
Satisfied	1, ref	1, ref		0, ref	0, ref		0, ref	0, ref	
Very satisfied	1.41 (1.03, 1.92)	**1.74** (1.17, 2.59)	0.006	0.04 (0.02, 0.06)	**0.02** (0.01, 0.04)	0.001	5.75 (3.18, 8.33)	**4.21** (1.97, 6.45)	< 0.001
Information and attention the family and relatives had received									
Very dissatisfied/dissatisfied	0.78 (0.22, 2.74)	1.79 (0.40, 8.15)	0.449	–0.07 (–0.127, 0.002)	**–0.05** (–0.09, –0.00	0.034	–2.68 (–14.18, 8.82)	–0.66 (–10.91, 9.59)	0.899
Satisfied	1, ref	1, ref		0, ref	0, ref		0, ref	0, ref	
Very satisfied	1.34 (0.95, 1.89)	1.31 (0.83, 2.06)	0.247	0.02 (–0.00, 0.04)	0.01 (–0.00, 0.03)	0.160	3.30 (0.44, 6.16)	1.86 (–0.65, 4.36)	0.147
Attention received from the staff									
Very dissatisfied/dissatisfied	NA	NA		0.20 (–0.03, 0.42)	0.05 (–0.12, 0.22)	0.591	31.53 (–31.46, 94.51)	11.37 (–5.14, 27.8)	0.177
Satisfied	1, ref	1, ref		0, ref	0, ref		0, ref	0, ref	
Very satisfied	1.98 (1.15, 3.38)	1.63 (0.77, 3.44)	0.202	0.06 (0.03, 0.08)	**0.03** (0.01, 0.05)	0.007	6.74 (1.94, 11.53)	**4.42** (0.62, 8.2)	0.023
Cooperation with the staff									
Very dissatisfied/dissatisfied	NA	NA		–0.14 (–0.28, –0.01)	–0.11 (–0.24, 0.02)	0.107	–34.84 (–62.82, –6.86)	**–30.73** (–56.99, –4.46)	0.022
Satisfied	1, ref	1, ref		0, ref	0, ref		0, ref	0, ref	
Very satisfied	2.48 (1.54, 4.01)	**3.28** (1.64, 6.56)	0.001	0.05 (0.02, 0.08)	**0.03** (0.01, 0.05)	0.002	5.76 (2.05, 9.47)	**5.06** (1.80 8.32)	0.002
Information given on where to get more support after discharge									
Very dissatisfied/dissatisfied	1.00 (0.43, 2.33)	0.75 (0.27, 2.10)	0.585	–0.02 (–0.07, 0.03)	–0.00 (–0.05, 0.04)	0.879	–5.04 (–12.36, 2.28)	–3.61 (–10.76 3.53)	0.321
Satisfied	1, ref	1, ref		0, ref	0, ref		0, ref	0, ref	
Very satisfied	1.57 (1.14, 2.16)	**1.58** (1.05, 2.37)	0.028	0.02 (0.00, 0.03)	0.01 (–0.01, 0.02)	0.249	3.67 (1.04, 6.31)	2.09 (–0.21, 4.39)	0.075
Follow-up									
Rehabilitation process									
Very dissatisfied/dissatisfied	0.21 (0.06, 0.70)	0.57 (0.15, 2.18)	0.409	–0.11 (–0.15, –0.07)	**–0.09** (–0.13, –0.04)	0.001	–13.84 (–19.83, –7.84)	**–8.95** (–14.93, –2.98)	0.003
Satisfied	1, ref	1, ref		0, ref	0, ref		0, ref	0, ref	
Very satisfied	1.75 (1.29, 2.38)	**1.54** (1.07, 2.22)	0.020	0.03 (0.02, 0.05)	**0.02** (0.00, 0.03)	0.033	6.46 (3.94, 8.97)	**5.03** (2.72, 7.35)	< 0.001
Patient’s influence over the rehabilitation process									
Very dissatisfied/dissatisfied	0.42 (0.18, 0.99)	0.90 (0.34, 2.40)	0.833	–0.10 (–0.14, –0.07)	**–0.06** (–0.10, –0.02)	0.003	–14.42 (–20.59, –8.26)	**–9.24** (–15.41, –3.08)	0.003
Satisfied	1, ref	1, ref		0, ref	0, ref		0, ref	0, ref	
Very satisfied	1.76 (1.30, 2.40)	**1.64** (1.13, 2.36)	0.009	0.03 (0.02, 0.05)	**0.02** (0.01, 0.03)	0.006	6.03 (3.58, 8.47)	**4.00** (1.78, 6.22)	< 0.001
Information given concerning the stroke									
Very dissatisfied/dissatisfied	0.27 (0.12, 0.58)	**0.35** (0.14, 0.87	0.024	–0.07 (–0.10, –0.03)	**–0.04** (–0.07, –0.01)	0.021	–7.29 (–12.77, –1.81)	–4.14 (–9.26, 0.98)	0.113
Satisfied	1, ref	1, ref		0, ref	0, ref		0, ref	0, ref	
Very satisfied	1.40 (1.03, 1.90)	1.30 (0.90, 1.86)	0.164	0.02 (0.00, 0.03)	0.01 (–0.00, 0.02)	0.193	5.20 (2.67, 7.73)	**3.13** (0.85, 5.40)	0.007
Information and attention the family and relatives had received									
Very dissatisfied/dissatisfied	0.68 (0.34, 1.36)	0.69 (0.28, 1.66)	0.403	–0.04 (–0.08, –0.00)	0.00 (–0.04, 0.04)	0.997	–5.49 (–11.69, 0.70)	–1.22 (–6.87, 4.44)	0.673
Satisfied	1, ref	1, ref		0, ref	0, ref		0, ref	0, ref	
Very satisfied	1.89 (1.38, 2.60)	**1.67** (1.14, 2.44	0.008	0.04 (0.02, 0.05)	**0.02** (0.01, 0.03)	0.007	6.48 (3.86, 9.10)	**4.24** (1.90, 6.58)	< 0.001

a OR calculated by logistic regression, dichotomization Satisfied (scores 5–6) vs Not satisfied (scores 1–4).

b Coefficients calculated by linear regression.

NA: not applicable, OR: odds ratio, CI: confidence interval, ref: reference. Items shown in bold denote statistically significant results with *p*-value less than 0.05.

### Effect modification by sex and age on the associations between patient satisfaction and life satisfaction and health-related quality of life on discharge and at 1-year follow-up

Table IV displays the impact of sex on patient satisfaction in the regression models. On discharge, men satisfied with the rehabilitation (3 of 7 items) were more likely to be satisfied with LS and reported better HRQoL. These associations between patient satisfaction and LS declined at the 1-year follow-up. Women dissatisfied with the rehabilitation process and dissatisfied with the cooperation with staff were associated with poorer HRQoL on discharge. At the 1-year follow-up, the associations between patient satisfaction and HRQoL were more equally present between the sexes.

**Table IV:** Regression models of patient satisfaction and associations with Life Satisfaction (life as a whole) and Health-Related Quality of Life (EQ-5D), and effect modification of sex on discharge and at 1-year follow-up

Item	Life as a whole^a^		EQ-5D Index^b^		EQ VAS^b^	
	Multivariable adjusted, OR (95% CI)		Multivariable adjusted, Coefficient (95% CI)		Multivariable adjusted, Coefficient (95% CI)	
	Men	Women	Men	Women	Men	Women
Discharge						
Rehabilitation process						
Very dissatisfied/dissatisfied	NA	NA	NS	**–0.19** (–0.21, –0.17) *p* < 0.001	NS	**–43.11** (–46.46, –39.75) *p* < 0.000
Satisfied	1, ref	NS	0, ref	NS	0, ref	NS
Very satisfied	**2.21** (1.28, 3.83) *p* = 0.005	NS	**0.02** (0.00, 0.04) *p* = 0.023	NS	**3.90** (0.97, 6.83) *p* = 0.009	NS
Patient’s influence over the rehabilitation process						
Very dissatisfied/dissatisfied	NS	NA	NS	NS	NS	NS
Satisfied	1, ref	NS	0, ref	NS	0, ref	NS
Very satisfied	NS	NS	**0.02** (0.00, 0.03) *p* = 0.035	NS	**3.25** (0.49, 6.00) *p* = 0.021	NS
Information given concerning the stroke						
Very dissatisfied/dissatisfied	NS	NS	**0.04** (0.00, 0.08) *p* = 0.033	NS	NS	NS
Satisfied	1, ref	NS	0, ref	NS	0, ref	NS
Very satisfied	**1.95** (1.18, 3.23) *p* = 0.009	NS	**0.03** (0.01, 0.04) *p* = 0.004	NS	**3.10** (0.31, 5.88) *p* = 0.030	NS
Information and attention the family and relatives had received						
Very dissatisfied/dissatisfied	NS	NS	NS	NS	NS	Ns
Satisfied	1, ref	NS	0, ref	NS	0, ref	NS
Very satisfied	NS	NS	**0.02** (0.00, 0.04) *p* = 0.014	NS	NS	NS
Attention received from the staff						
Very dissatisfied/dissatisfied	NA	NA	NS	NA	**11.23** (5.8, 16.65) *p* = 0.001	NA
Satisfied	1, ref	NS	0, ref	NS	0, ref	NS
Very satisfied	NS	NS	NS	NS	**4.26** (0.23, 8.30) *p* = 0.039	NS
Cooperation with the staff						
Very dissatisfied/dissatisfied	NA	NA	NA	NS	NA	**–33.04** (–56.33, –6.76) *p* = 0.014
Satisfied	1, ref	NS	0, ref	NS	0, ref	NS
Very satisfied	**3.05** (1.43, 6.48) *p* = 0.004	**2.56** (1.16, 5.66) *p* = 0.02	**0.03** (0.00, 0.05) *p* = 0.020	NS	**4.55** (0.96, 8.15) *p* = 0.013	NS
Information given on where to get more support after discharge						
Very dissatisfied/dissatisfied	NS	NS	NS	NS	NS	NS
Satisfied	1, ref	NS	0, ref	NS	0, ref	NS
Very satisfied	**1.68** (1.00, 2.81) *p* = 0.0497	NS	NS	NS	NS	NS
Follow-up						
Rehabilitation process						
Very dissatisfied/dissatisfied	NS	NS	**–0.09** (–0.14, –0.04) *p* = 0.009	**–0.10** (–0.18, –0.02) *p* = 0.020	**–10.06** (–18.67, –1.44) *p* = 0.022	**–9.29** (–14.75, –3.84) *p* < 0.001
Satisfied	1, ref	NS	0, ref	NS	0, ref	NS
Very satisfied	NS	NS	NS	NS	**3.71** (0.66, 6.76) *p* = 0.017	**4.74** (1.51, 7.97) *p* = 0.004
Satisfied	1, ref	NS	0, ref	**–0.03** (–0.05, 0.00) *p* = 0.024	0, ref	NS
Very satisfied	NS	**1.76** (1.08, 2.87) *p* = 0.024	NS	NS	NS	**3.84** (0.83, 6.85) *p* = 0.012
Information given concerning the stroke						
Very dissatisfied/dissatisfied	NS	**0.09** (0.01, 0.74) *p* = 0.025	NS	NS	NS	**–9.65** (–17.32, –1.99) *p* = 0.014
Satisfied	1, ref	NS	0, ref	NS	0, ref	NS
Very satisfied	NS	NS	NS	NS	NS	**3.84** (0.83, 6.86) *p* = 0.013
Information and attention the family and relatives had received						
Very dissatisfied/dissatisfied	NS	NS	NS	NS	NS	NS
Satisfied	1, ref	**0.40** (0.21, 0.74) *p* = 0.004	0, ref	NS	0, ref	NS
Very satisfied	NS	NS	**0.02** (0.00, 0.04) *p* = 0.047	NS	**3.48** (0.46, 6.50) *p* = 0.024	**4.28** (1.03, 7.54) *p* = 0.010

a OR calculated by logistic regression, dichotomisation Satisfied (scores 5–6) vs Not satisfied (scores 1–4).

b Coefficients calculated by linear regression.

NA: not applicable, OR: odds ratio, CI: confidence interval, ref: reference. Items shown in bold denote statistically significant results with *p*-value less than 0.05.

Table V presents the impact of age on patient satisfaction in the regression models. Younger patients who were dissatisfied with the rehabilitation process reported poorer EQ Index, while older patients who were dissatisfied with the rehabilitation process reported lower values on EQ VAS. Furthermore, younger patients satisfied with various aspects of rehabilitation reported better HRQoL, while older patients who were satisfied with many aspects of the rehabilitation reported both better LS and HRQoL.

**Table V:** Regression models of patient satisfaction and associations with life satisfaction (life as a whole) and health-related quality of life (EQ-5D), and effect modification of age at discharge and 1-year follow-up

Item	Life as a whole^a^		EQ-5D Index^b^		EQ VAS^b^	
	Multivariable adjusted, OR (95% CI)		Multivariable adjusted, Coefficient (95% CI)		Multivariable adjusted, Coefficient (95% CI)	
	Below median age	Above median age	Below median age	Above median age	Below median age	Above median age
Discharge						
Rehabilitation process						
Very dissatisfied/dissatisfied	NA	NA	**–0.18** (–0.20, –0.16) *p* < 0.001	NS	**–29.99** (–54.36, –5.61) *p* = 0.016	**–27.09** (–30.92, –23.26) *p* < 0.001
Satisfied	1, ref	NS	0, ref	NS	0, ref	NS
Very satisfied	**1.84** (1.03, 3.29) *p* = 0.039	**2.61** (1.41, 4.83) *p* = 0.002	**0.02** (0.00, 0.04) *p* = 0.015	**0.03** (0.01, 0.05) *p* = 0.009	**5.44** (2.31, 8.57) *p* < 0.001	**4.45** (1.03, 7.87) *p* = 0.011
Patient’s influence over the rehabilitation process						
Very dissatisfied/dissatisfied	NS	NA	NS	NS	NS	NS
Satisfied	1, ref	NS	0, ref	NS	0, ref	NS
Very satisfied	NS	NS	NS	NS	NS	NS
Information given concerning the stroke						
Very dissatisfied/dissatisfied	NS	NS	NS	NS	NS	NS
Satisfied	1, ref	NS	0, ref	NS	0, ref	NS
Very satisfied	**1.76** (1.04, 2.97) *p* = 0.038	**2.33** (1.28, 4.27) *p* = 0.006	**0.03** (0.01, 0.05) *p* = 0.002	**0.03** (0.01, 0.05) *p* = 0.004	**3.90** (0.99, 6.82) *p* = 0.001	**3.74** (0.59, 6.88) *p* = 0.020
Information and attention the family and relatives had received						
Very dissatisfied/dissatisfied	NS	NS	**–0.05** (–0.10, –0.01) *p* = 0.030	**0.03** (0.00, 0.06) *p* = 0.049	NS	Ns
Satisfied	1, ref	NS	0, ref	NS	0, ref	NS
Very satisfied	NS	NS	NS	NS	NS	NS
Attention received from the staff						
Very dissatisfied/dissatisfied	NA	NA	NS	**0.08** (0.04, 0.11) *p* < 0.001	NA	NS
Satisfied	1, ref	NS	0, ref	**0.06** (0.01, 0.10) *p* = 0.015	0, ref	NS
Very satisfied	NS	NS	**0.05** (0.02. 0.09) *p* = 0.001	**0.06** (0.02, 0.09) *p* = 0.015	**5.29** (0.01, 10.57) *p* = 0.049	NS
Cooperation with the staff						
Very dissatisfied/dissatisfied	NA	NA	NS	NA	**–28.92** (–55.67, –2.17) *p* = 0.034	NA
Satisfied	1, ref	NS	0, ref	0.05** (0.02, 0.09)	0, ref	NS
Very satisfied	**3.82** (1.42, 10.29) *p* = 0.008	**4.64** (1.71, 12.60) *p* = 0.003	**0.06** (0.03, 0.08) *p* < 0.001	**0.06** (0.03, 0.08) *p* < 0.001	**7.19** (02.81, 11.58) *p* = 0.001	**5.72** (1.25, 10.19) *p* = 0.012
Information given on where to get more support after discharge						
Very dissatisfied/dissatisfied	NS	NS	NS	NS	NS	NS
Satisfied	1, ref	NS	0, ref	NS	0, ref	NS
Very satisfied	NS	**2.33** (1.23, 4.39) *p* = 0.009	NS	NS	NS	NS
Analysis for follow-up						
Rehabilitation process						
Very dissatisfied/dissatisfied	NS	NS	**–0.09** (–0.15, –0.03) *p* = 0.002	**–0.07** (–0.14, –0.01) *p* = 0.028	NS	**–8.86** (–15.61, –2.11) *p* = 0.010
Satisfied	1, ref	NS	0, ref	NS	0, ref	NS
Very satisfied	NS	**2.27** (1.35, 3.80) *p* = 0.002	**0.02** (0.00, 0.04) *p* = 0.030	NS	**6.57** (3.33, 9.81) *p* < 0.001	**6.76** (3.33, 10.19) *p* < 0.001
Patient’s influence over the rehabilitation process						
Very dissatisfied/dissatisfied	NS	NA	NS	**–0.07** (–0.13, –0.01) *p* = 0.015	NS	**–7.83** (–14.56, –1.11) *p* = 0.022
Satisfied	1, ref	NS	0, ref	0, ref	0, ref	NS
Very satisfied	**1.87** (1.14, 3.06) *p* = 0.014	**2.23** (1.33, 3.73) *p* = 0.002	**0.02** (0.00, 0.04) *p* 0.027	NS	**5.56** (2.62, 8.50) *p* < 0.001	**4.85** (1.67, 8.03) *p* = 0.003
Information given concerning the stroke						
Very dissatisfied/dissatisfied	NS	NS	NS	NS	NS	NS
Satisfied	1, ref	NS	0, ref	NS	0, ref	NS
Very satisfied	NS	**1.78** (1.08, 2.92) *p* = 0.023	NS	NS	**3.69** (0.58, 6.80) *p* = 0.020	**3.52** (0.47, 6.58) *p* = 0.024
Information and attention the family and relatives had received						
Very dissatisfied/dissatisfied	NS	NS	NS	NS	NS	NS
Satisfied	1, ref	**1.92** (1.11, 3.32) p = 0.020	0, ref	NS	0, ref	NS
Very satisfied	**2.16** (1.30, 3.59) *p* = 0.003	**2.35** (1.38, 4.01) *p* = 0.002	**0.02** (0.00, 0.04) *p* = 0.017	**0.02** (0.00, 004) *p* = 0.044	**5.79** (2.56, 9.01) *p* < 0.001	**5.37** (2.02, 8.73) *p* = 0.002

a OR calculated by logistic regression, dichotomization Satisfied (scores 5–6) vs Not satisfied (scores 1–4).

b Coefficients calculated by linear regression.

NA: not applicable, OR: odds ratio, CI: confidence interval, ref: reference. Items shown in bold denote statistically significant results with *p*-value less than 0.05.

## DISCUSSION

This study investigated the experiences and effects of hospital-based outpatient rehabilitation among 1,068 stroke survivors, using a PREM to assess patient satisfaction and PROMs to evaluate LS and HRQoL. Most participants were satisfied with several aspects of outpatient rehabilitation on discharge and at the 1-year follow-up. Stroke survivors with higher LS scores and higher HRQoL values tended to report greater satisfaction with their outpatient rehabilitation.

Previous studies have shown that higher functional levels (24) and returning to work (34) were linked to greater satisfaction with rehabilitation. In line with this, our results indicated that greater satisfaction with the rehabilitation process was related to better LS and HRQoL both on discharge and at the follow-up. Dissatisfaction, on the other hand, appeared to be linked with poorer HRQoL at both time points, which might reflect the importance of ongoing support to help maintain health-related functioning. Higher values of HRQoL observed on discharge seemed to be sustained at follow-up, which may indicate that discharge could represent an important point in the rehabilitation trajectory, although this needs to be further explored.

The patients’ influence over the rehabilitation process was associated with LS and HRQoL at the 1-year follow-up but not on discharge. Earlier research has suggested that patients’ participation and involvement in care are decisive factors for satisfaction, sometimes outweighing disease-specific factors after stroke (35). Satisfaction with care has also been identified as a key indicator of quality of life (36). Moreover, dissatisfaction with healthcare 1-year post-stroke has been associated with low coping skills, impaired function, and limited participation (37). In our cohort, patients who were very satisfied with the information provided concerning stroke on discharge also tended to report better LS and HRQoL, which may indicate the value of patient-centred care (38) and provision of repeated information (39, 40). In contrast, satisfaction with the information and attention given to the family and relatives was not associated with the patients’ self-reported LS and HRQoL on discharge.

The sex differences observed in our study were somewhat inconsistent and therefore difficult to interpret. However, women were somewhat more dissatisfied with the rehabilitation process both on discharge and at the 1-year follow-up compared with men. This finding is in line with earlier research showing that women post-stroke generally report poorer HRQoL than men (19). Women also tend to be older at stroke onset and more severely affected by the stroke (19, 41–44). In our predominantly working-age sample, these explanations may be less relevant. One alternative explanation for our sample could be related to family and caregiving responsibilities, but this remains speculative because we did not include data on family roles or caregiving burden. Recent qualitative work has further highlighted that younger women may face unique challenges in resuming social and family roles after stroke, which could negatively influence their satisfaction with rehabilitation (25).

Age appeared to play some role in the associations observed. In our data, the relationship between patient satisfaction and LS was somewhat stronger in older patients, whereas the association with HRQoL seemed more evident among younger patients. This may indicate that different age groups value different aspects of their rehabilitation, although the data do not permit firm conclusions on this matter. A previous study has reported low LS among younger stroke survivors (45). Although age-related differences in LS and HRQoL have been described, findings remain inconsistent across studies (35). Additionally, Ullberg et al. identified a higher prevalence of stroke-related health problems among younger patients, many of whom experience invisible impairments that affect everyday life (46). Such factors may partly explain why the younger stroke survivors in our study reported lower HRQoL despite associations between satisfaction and HRQoL outcomes in this group.

### Methodological considerations

This study focused on individuals who received hospital-based outpatient rehabilitation, excluding those who received inpatient care. This may have introduced selection bias and reduced the generalizability of the results, especially for older adults or those with more severe impairments. While adherence to national stroke guidelines was assumed, differences in rehabilitation volume (i.e., hours per week) and duration (i.e., total days/weeks) as well as access to multidisciplinary teams likely occurred. The study did not consider specific rehabilitation content or differences between units, which could have influenced outcomes. Furthermore, the lack of socioeconomic and disability-related data limits the ability to explain variations in satisfaction and HRQoL. Decreasing response rates over time may have led to recall bias and missing data, affecting internal validity. Despite a smaller sample size, long-term follow-up was prioritized. Nevertheless, the study’s national scope, with data from rehabilitation units across Sweden, enhances generalizability. The use of a large, consecutively registered sample and valid PROMs and PREM collected at defined timepoints provide valuable longitudinal insights into patient experiences and outcomes.

In conclusion, the findings suggest that while stroke survivors generally report satisfaction with hospital-based outpatient rehabilitation, certain outcomes may change over time, with variations across age and sex. The sustained improvements in HRQoL observed from discharge to follow-up suggest discharge as an important time point for supporting patients and consolidating rehabilitation gains. This finding underscores the clinical importance of structured discharge planning, goal-setting, and continued follow-up. The observed variations depending on sex and age highlight the potential value of tailoring rehabilitation strategies to meet the needs of specific subgroups of patients. Overall, the study illustrates the value of national register data incorporating patient-reported outcomes and experiences, contributing to development of more person-centred and individualized outpatient stroke rehabilitation.

## Supplementary Material


