# Supplementary material for: PATIENT SATISFACTION WITH HOSPITAL-BASED OUTPATIENT REHABILITATION AFTER STROKE IN SWEDEN AND ITS ASSOCIATION WITH LIFE SATISFACTION AND HEALTH-RELATED QUALITY OF LIFE: A LONGITUDINAL NATIONAL REGISTER STUDY
Source: J Rehabil Med. 2026 Jan 14;58:43966. doi: 10.2340/jrm.v58.43966 (PMC12813626; doi:10.2340/jrm.v58.43966)
Supplement: Supplementary file 1 [file JRM-58-43966-s1.pdf]

Supplementary material has been published as submitted. It has not been copyedited, or typeset by Acta Dermato-Venereologica

Table SI. Capture of the different instruments from admission, discharge and 1-year follow-up, separately for gender

	Admission		Discharge		Follow-up	
	Women	Men	Women	Men	Women	Men
	N=417	N=651	N=417	N=651	N=417	N=651
EQ-5D						
Yes, by patient	367 (88.0%)	568 (87.3%)	338 (81.1%)	493 (75.7%)	407 (97.6%)	633 (97.2%)
Cannot be conducted	6 (1.4%)	9 (1.4%)	3 (0.7%)	16 (2.5%)	1 (0.2%)	1 (0.2%)
No	44 (10.6%)	74 (11.4%)	76 (18.2%)	142 (21.8%)	1 (0.2%)	8 (1.2%)
Missing					8 (1.9%)	9 (1.4%)
LiSat-11 (global item)						
Yes, by patient	261 (62.6%)	401 (61.6%)	297 (71.2%)	440 (67.6%)	339 (81.3%)	538 (82.6%)
Cannot be conducted			2 (0.5%)	17 (2.6%)	5 (1.2%)	1 (0.2%)
No	156 (37.4%)	250 (38.4%)	118 (28.3%)	194 (29.8%)	65 (15.6%)	103 (15.8%)
Missing					8 (1.9%)	9 (1.4%)
Patient satisfaction						
Yes, by patient			355 (85.1%)	541 (83.1%)	352 (84.4%)	547 (84.0%)
Cannot be conducted			2 (0.5%)	15 (2.3%)	2 (0.5%)	1 (0.2%)
No			54 (12.9%)	83 (12.7%)	51 (12.2%)	92 (14.1%)
Do not know			6 (1.4%)	12 (1.8%)	4 (1.0%)	2 (0.3%)
Missing	417 (100.0%)	651 (100.0%)			8 (1.9%)	9 (1.4%)

EQ-5D: EuroQol five dimensions questionnaire; LiSat: Life Satisfaction

Table SII. Crude data of EQ-5D for admission, discharge and 1-year follow up, separately per gender

	Admission		Discharge		Follow-up	
	Women	Men	Women	Men	Women	Men
	N=367	N=568	N=338	N=493	N=407	N=633
EQ VAS	median=60.0 (IQR 50.0-70.0)	median=65.0 (IQR 50.0-80.0)	median=70.0 (IQR 57.0-80.0)	median=75.0 (IQR 60.0-80.5)	median=70.0 (IQR 53.5-80.0)	median=70.0 (IQR 60.0-80.0)
EQ VAS	mean=59.3 (SD 16.9)	mean=63.0 (SD 19.4)	mean=67.7 (SD 18.3)	mean=71.0 (SD 17.4)	mean=66.7 (SD 19.0)	mean=68.8 (SD 18.7)
Mobility						
One (1)	223 (60.8%)	319 (56.2%)	228 (67.5%)	316 (64.1%)	284 (69.8%)	417 (65.9%)
Two (2)	140 (38.1%)	245 (43.1%)	110 (32.5%)	173 (35.1%)	122 (30.0%)	211 (33.3%)
Three (3)	4 (1.1%)	4 (0.7%)	0 (0.0%)	4 (0.8%)	1 (0.2%)	5 (0.8%)
Self-care						
One (1)	309 (84.2%)	478 (84.2%)	303 (89.6%)	431 (87.4%)	358 (88.0%)	556 (87.8%)
Two (2)	50 (13.6%)	76 (13.4%)	30 (8.9%)	49 (9.9%)	39 (9.6%)	58 (9.2%)
Three (3)	8 (2.2%)	14 (2.5%)	5 (1.5%)	13 (2.6%)	10 (2.5%)	19 (3.0%)
Usual activitites						
One (1)	90 (24.5%)	213 (37.5%)	136 (40.2%)	254 (51.5%)	170 (41.8%)	323 (51.0%)
Two (2)	201 (54.8%)	258 (45.4%)	158 (46.7%)	192 (38.9%)	189 (46.4%)	242 (38.2%)
Three (3)	76 (20.7%)	97 (17.1%)	44 (13.0%)	47 (9.5%)	48 (11.8%)	68 (10.7%)
Pain/discomfort						
One (1)	84 (22.9%)	215 (37.9%)	100 (29.6%)	207 (42.0%)	123 (30.2%)	255 (40.3%)
Two (2)	254 (69.2%)	321 (56.5%)	207 (61.2%)	269 (54.6%)	248 (60.9%)	343 (54.2%)
Three (3)	29 (7.9%)	32 (5.6%)	31 (9.2%)	17 (3.4%)	36 (8.8%)	35 (5.5%)
Anxiety/depression						
One (1)	134 (36.5%)	283 (49.8%)	158 (46.7%)	299 (60.6%)	168 (41.3%)	320 (50.6%)
Two (2)	211 (57.5%)	261 (46.0%)	168 (49.7%)	185 (37.5%)	218 (53.6%)	290 (45.8%)
Three (3)	22 (6.0%)	24 (4.2%)	12 (3.6%)	9 (1.8%)	21 (5.2%)	23 (3.6%)

EQ-5D: EuroQol five dimensions questionnaire.

Table SIII. Crude data of Life Satisfaction (Life as a whole) instrument for admission, discharge, and 1-year follow-up, separately for gender

	Admission		Discharge		Follow-up	
	Women	Men	Women	Men	Women	Men
	N=261	N=401	N=297	N=440	N=339	N=538
Global item ”Life as whole”						
1	4 (1.5%)	9 (2.2%)	5 (1.7%)	4 (0.9%)	8 (2.4%)	16 (3.0%)
2	20 (7.7%)	28 (7.0%)	14 (4.7%)	20 (4.5%)	18 (5.3%)	30 (5.6%)
3	56 (21.5%)	73 (18.2%)	37 (12.5%)	44 (10.0%)	53 (15.6%)	76 (14.1%)
4	97 (37.2%)	143 (35.7%)	116 (39.1%)	158 (35.9%)	142 (41.9%)	197 (36.6%)
5	65 (24.9%)	116 (28.9%)	98 (33.0%)	173 (39.3%)	91 (26.8%)	178 (33.1%)
6	19 (7.3%)	32 (8.0%)	27 (9.1%)	41 (9.3%)	27 (8.0%)	41 (7.6%)

Table SIV. Crude data of Patient satisfaction for admission, discharge, and 1-year follow-up, separately for gender.

	Discharge		Follow-up	
	Women	Men	Women	Men
	N=355	N=541	N=352	N=547
The rehabilitation process				
Very dissatisfied			3 (0.9%)	1 (0.2%)
Dissatisfied	3 (0.8%)	3 (0.6%)	12 (3.4%)	30 (5.5%)
Satisfied	83 (23.4%)	160 (29.6%)	138 (39.2%)	224 (41.0%)
Very satisfied	262 (73.8%)	377 (69.7%)	187 (53.1%)	278 (50.8%)
Missing	7 (2.0%)	1 (0.2%)	12 (3.4%)	14 (2.6%)
The patient's influence over the rehabilitation process				
Very dissatisfied			4 (1.1%)	4 (0.7%)
Dissatisfied	5 (1.4%)	6 (1.1%)	14 (4.0%)	26 (4.8%)
Satisfied	108 (30.4%)	208 (38.4%)	148 (42.0%)	277 (50.6%)
Very satisfied	236 (66.5%)	319 (59.0%)	162 (46.0%)	208 (38.0%)
Missing	6 (1.7%)	8 (1.5%)	24 (6.8%)	32 (5.9%)
The information given about the stroke				
Very dissatisfied	1 (0.3%)	2 (0.4%)	5 (1.4%)	3 (0.5%)
Dissatisfied	9 (2.5%)	16 (3.0%)	24 (6.8%)	41 (7.5%)
Satisfied	144 (40.6%)	239 (44.2%)	170 (48.3%)	278 (50.8%)
Very satisfied	187 (52.7%)	269 (49.7%)	139 (39.5%)	204 (37.3%)
Missing	14 (3.9%)	15 (2.8%)	14 (4.0%)	21 (3.8%)
The information and attention the family and relatives had received				
Very dissatisfied	2 (0.6%)	0 (0.0%)	4 (1.1%)	4 (0.7%)
Dissatisfied	5 (1.4%)	7 (1.3%)	19 (5.4%)	33 (6.0%)
Satisfied	96 (27.0%)	169 (31.2%)	137 (38.9%)	242 (44.2%)
Very satisfied	194 (54.6%)	300 (55.5%)	143 (40.6%)	216 (39.5%)
Missing	58 (16.3%)	65 (12.0%)	49 (13.9%)	52 (9.5%)
The attention received from the staff				
Very dissatisfied	0 (0.0%)	1 (0.2%)		
Dissatisfied				
Satisfied	36 (10.1%)	53 (9.8%)		
Very satisfied	316 (89.0%)	487 (90.0%)		

Missing	3 (0.8%)	0 (0.0%)		
The cooperation with the staff				
Very dissatisfied				
Dissatisfied	3 (0.8%)	0 (0.0%)		
Satisfied	44 (12.4%)	78 (14.4%)		
Very satisfied	304 (85.6%)	463 (85.6%)		
Missing	4 (1.1%)	0 (0.0%)		
The information given om where to get more support after discharge				
Very dissatisfied	1 (0.3%)	4 (0.7%)		
Dissatisfied	9 (2.5%)	20 (3.7%)		
Satisfied	143 (40.3%)	206 (38.1%)		
Very satisfied	167 (47.0%)	262 (48.4%)		
Missing	35 (9.9%)	49 (9.1%)		
